# DG-RAR for the treatment of symptomatic grade III and grade IV haemorrhoids: a 12-month multi-centre, prospective observational study

**DOI:** 10.1007/s10353-012-0182-8

**Published:** 2013-02-09

**Authors:** S. Roka, D. Gold, P. Walega, S. Lancee, E. Zagriadsky, A. Testa, A. N. Kukreja, A. Salat

**Affiliations:** 1 Department of Surgery, Medical University of Vienna, Währinger Gürtel 18-20, 1090 Vienna, Austria; 2 Professorial Department of Surgery, St Vincents Clinical School, University of New South Wales, Sydney, Australia; 3 Department of Surgery, North Hampshire Hospital, Basingstoke, England; 4 3rd Department of Surgery, Collegium Medicum, Jagiellonian University, Pradnicka Str. 37, 31-202 Krakow, Poland; 5 Department of Surgery, Landeskrankenhaus Bludenz, Spitalgasse 13, 6700 Bludenz, Austria; 6 Medical Coloproctology Center, Bolshaya Molchanovka Street, Building 32, 121069 Moscow, Russia; 7 Department of Surgery, General Surgery Unit, Ospedale San Pietro Fatebenefratelli, Via Cassia 600, 00189 Rome, Italy; 8 Ratandeep Surgical Hospital and Endoscopy Clinic, Nakshatra 2nd Floor, Ganesh Gali, Maninagar Cross Roads, 380008 Ahmedabad, Gujarat India

**Keywords:** Haemorrhoids, Haemorrhoidal artery ligation, Recto-anal repair

## Abstract

**Background:**

Ultrasound-guided techniques represent a new treatment option in the treatment of haemorrhoids. Doppler-guided haemorrhoidal artery ligation (DG-HAL) proved efficacious in early haemorrhoidal disease, but lacks efficacy for stages III/IV. For these patients, haemorrhoidal artery ligation (HAL) has been combined with a running suture to reduce prolapsing haemorrhoidal tissue (recto-anal repair (RAR)).

**Methods:**

A prospective observational study was conducted in 184 patients with grade III (58 %) or grade IV (42 %) haemorrhoids in seven coloproctological centres. Primary endpoints were the recurrence of symptoms and need of further treatment (medical or surgical).

**Results:**

Post-operative complications were seen in 8 % of patients. After a follow-up of 3 months, 91 % of patients were free of symptoms and 91 % of patients were satisfied with the result. After a follow-up of 12 months, 89 % of patients were free of symptoms and 88 % were satisfied with the result. Nineteen per cent of patients received further medical or surgical treatment.

**Conclusions:**

Doppler-guided recto-anal repair (DG-RAR) proves to be an effective treatment option for the treatment of advanced haemorrhoidal disease that shows equal results to other established treatment options.

## Introduction

A variety of treatment options are available for haemorrhoidal disease. Whereas early stages of the disease can usually be treated conservatively with success, advanced stages require a surgical approach. At present, surgical treatment generally involves resection of the haemorrhoidal cushions, as in conventional haemorrhoidectomy (CH) or prolapse reduction as in stapled haemorrhoidopexy (SH) [1]. Despite excellent long-term results, CH is associated with significant side effects and complications. In addition to excessive post-operative pain, symptoms of incontinence and anal stenosis have been described in considerable numbers [2–5]. For SH, results are more convenient concerning post-operative pain. However, reoperations have been reported in up to 10 % of patients [5, 6] and, albeit very rare, this technique can be associated with severe septic complications [5, 7].

Minimally invasive treatment options have become popular over the last few decades, even in coloproctology. Doppler-guided haemorrhoidal artery ligation (DG-HAL) was first described in 1995 by Morinaga et al. [8]. The principle of the technique was to ligate the proximal submucosal haemorrhoidal arteries, thereby reducing the blood flow to the haemorrhoidal cushions and eventually leading to fibrosis and shrinking of the haemorrhoids. Haemorrhoidal artery ligation (HAL) has since been analysed in several studies, which show excellent results for grade II and III haemorrhoidal disease. However, while HAL appears to control pain, bleeding, pruritus and mucous discharge with success, control of the prolapse seems to be more difficult in patients suffering from grade III or grade IV haemorrhoids. Persistent or recurrent prolapse in grade III disease has been described with rates of 6 [9], 9 [10] and 14 % [11]. In studies involving patients with grade IV disease, even higher recurrence rates (RRs) such as 24 [10], 59 [11] and 67 % [9] have been observed.

For these patients, in particular those suffering from grade IV haemorrhoids, reduction of inflow alone seems insufficient to reduce the haemorrhoidal cushions. Recto-anal repair (RAR), a technique developed and described by Scheyer [12], addresses this problem by supplementing HAL with a further step, whereby the haemorrhoidal tissue is gathered up and lifted back into position. Based on this principle of tissue reduction, RAR is an alternative to surgical removal that is made possible by placing a longitudinal running suture while using a specially designed proctoscope.

The aim of this prospective observational study for grade III and IV haemorrhoidal disease was to review several technical aspects and analyze 1-year results of this new method (DG-HAL/RAR) in terms of recurrence of prolapse and symptoms other than prolapse.

## Patients and methods

This study was designed as a prospective observational study and was approved by the institutional review board. Included in the study were patients between the age of 18 and 80 years with symptomatic grade III and IV haemorrhoidal disease (according to Goligher classification), who had given their informed consent. Patients with a history of prior anal surgery and those considered unfit for surgery were excluded. Pregnant women and those in the puerperium were also excluded. Prior to surgery, all patients underwent a detailed examination for symptoms of haemorrhoidal disease. A standardised form was used to collect data from each patient during the study period.

Between March 2007 and December 2007, a total of 184 patients entered the study in seven proctological institutions. The centres contributed a median number of 21 patients (range 5–83). Patients were not required to enter the study in a consecutive order and the total number of patients screened was not documented. Of those 184 patients, 124 were male (65 %) and 64 female (35 %). The median age was 46.8 years (range 23–76). The number of patients suffering from grade III haemorrhoids was 107 (58 %), while 77 patients (42 %) were classified to suffer from grade IV haemorrhoids. Pain was reported by 136 patients (74 %), itching by 139 (76 %) and bleeding by 174 (95 %).

The surgical procedure was performed using the DG-RAR proctoscope (A.M.I. Agency for Medical Innovations GmbH) according to the manufacturer’s guidelines. The proctoscope consists of an outer and inner tube. In the closed position, there is a lateral opening proximal to the ultrasound probe that allows detection and ligation of the feeding vessels (haemorrhoidal artery ligation). Rotating the outer tube of the proctoscope gradually opens a lateral slit to enable placement of a running suture, which serves to reduce the anal cushions and secure them back in their anatomical position (prolapse reduction). There were no recommendations for pain management. Post-operative pain was assessed using a visual analogue scale (VAS). Follow-up with a physical examination and questioning for recurrence of symptoms and satisfaction was performed 3 and 12 months after surgery by the treating physician.

Statistical analysis was performed using SPSS V 18.0.0. Median and mean were used for descriptive purposes. A linear regression analysis was performed to test influence of certain variables on recurrence of prolapse, recurrence of symptoms and patient satisfaction. A *p*-value of < 0.05 was considered significant.

## Results

### Surgical procedure

A median of six (2–11) ligations and three (range 1–9) prolapse-reduction sutures (PRSs) were performed, whereby 80 % of patients had six or more ligations, and 80 % of patients had three or more PRSs. Surgery was performed under general anaesthesia in 132 patients (72 %), spinal anaesthesia in 45 (25 %) and local anaesthesia in 7 (4 %). The mean operating time was 35 min (range 13–75). Instances of significant bleeding during the procedure were observed in three patients (2 %). Post-operative complications were seen in 14 patients (8 %). Among them, 2 patients (1 %) suffered from bleeding which required operative revision, 11 patients (7 %) had perianal thromboses and 1 patient (1 %) was diagnosed with unspecified proctitis. The mean duration of the hospital stay was 2.3 days (range 1–9).

### Post-operative pain

In the immediate post-operative period, i.e. prior to discharge from hospital, 20 % of the patients reported pain higher than VAS 4. This gradually diminished by day 4, where only 3 % of patients reported pain higher than VAS 4. Thirteen patients (7 %) reported pain higher than VAS 7 immediately after surgery. This gradually decreased to 1 % at day 4. There was only one patient with persistent pain for more than 4 days, post-operatively (Table 1).

**Table 1:** Burden of post-operative pain as measured by visual analogue scale (VAS ~ 1–10)

Time (day)	VAS £ 3 *n* (%)	VAS 4–6 *n* (%)	VAS ³ 7 *n* (%)	Total *n*
1	115 (74.2)	34 (21.9)	6 (3.9)	155
2	102 (79.8)	22 (17.2)	4 (3.1)	128
3	95 (84.8)	12 (10.7)	5 (4.5)	112
4	93 (85.3)	15 (13.7)	1 (0.9)	109

### Recurrence of symptoms

Three months follow-up was completed for all 184 patients (Table 2). Pain was still present in 14 patients (8 %), itching in 19 (10 %) and bleeding in 11 (6 %). Twelve patients (7 %) experienced persistent or recurrent prolapse. One hundred and sixty-seven patients (91 %) were free of symptoms and 168 patients (91 %) were overall satisfied with the result of the procedure. Of these patients, 28 (15 %) received further treatment including CH (1 patient, 1 %), excision of skin tags (12 patients, 7 %), sclerotherapy (5 patients, 3 %) and treatment with an ointment, undefined and by surgeon preference (10 patients, 5 %).

**Table 2:** Preoperative status compared with 3 and 12 months post-operative status

	Before surgery (*n* = 184; %)	3 months (*n* = 184; %)	12 months (*n* = 167; %)
Pain	136 (73.9)	14 (7.6)	10 (4.8)
Itching	139 (75.5)	19 (10.3)	9 (4.2)
Bleeding	174 (94.6)	11 (5.9)	13 (6.6)
Patient satisfaction		168 (91.3)	148 (87.6)
Prolapse	184 (100)	12 (6.5)	21 (11.4)
Symptom and prolapse free		162 (88)	141 (84.4)
Symptom free		167 (90.8)	152 (89.4)
Further treatment		28 (15.2)	32 (18.9)

At 12 month, follow-up was completed for 167 patients (92 %). Pain was still present in 10 of these patients (5 %), itching in 9 (4 %) and bleeding in 13 (7 %). Persistent or recurrent prolapse was experienced by 21 patients (11 %). One hundred and fifty-two patients (89 %) were free of symptoms and 148 patients (88 %) were satisfied with the result of the procedure. Thirty-two patients received one or more further treatments (19 %). These included CH (*n* = 2), excision of skin tags (*n* = 12), sclerotherapy (*n* = 5), rubber band ligation (*n* = 4) and treatment with an ointment, undefined and by surgeon preference (*n* = 13).

### Factors influencing outcome

Grade of disease, sex, age and the number of ligations and PRSs were tested for their respective influence on the recurrence of prolapse or symptoms, and patient satisfaction. With respect to recurrence of prolapse, there was a significant difference between patients with grade III and IV disease (Tables 3 and 4). The number of PRSs, and in particular the number of ligations, were shown to have significant influence on the recurrence of symptoms. With respect to symptom recurrence, optimal results were obtained with five to seven ligations (median 6) and three to four PRSs (median 4). The only factor correlating significantly with patient satisfaction was the number of ligations.

**Table 3:** Effectiveness of DG-RAR at 3 and 12 months, stratified for grade of haemorrhoidal disease

	3 months		12 months	
	Grade III (*n* = 107; %)	Grade IV (*n* = 77; %)	Grade III (*n* = 99; %)	Grade IV (*n* = 70; %)
Recurrence of prolapse	6 (6)	6 (8)	8 (8)	13 (18)
Pain	5 (5)	9 (12)	3 (3)	7 (10)
Itching	9 (8)	10 (13)	2 (2)	7 (10)
Bleeding	3 (3)	8 (10)	2 (2)	8 (11)
Patient satisfaction	88.8	94.7	87.0	88.4

**Table 4:** Multi-variate analysis of factors influencing the parameters of efficacy

	Recurrence of prolapse		Recurrence of symptoms		Patient satisfaction	
	RR (range)	*p*	RR (range)	*p*	RR (range)	*p*
Ligations	− 0.04 (− 0.83 to 0.16)	0.18	− 0.12 (− 0.17 to − 0.07)	0.0001	0.115 (0.06 to 0.17)	0.0001
Grade	0.14 (0.03 to 0.2)	0.01	0.63 (− 0.38 to 0.16)	0.221	− 0.22 (− 0.13 to 0.09)	0.69
Sex	− 0.07 (− 0.17 to 0.03)	0.17	− 0.93 (− 0.19 to 0.01)	0.053	− 0.19 (− 0.7 to 0.32)	0.46
Age	0.004 (0.0 to 0.01)	0.057	0.002 (− 0.003 to 0.00)	0.474	0.02 (− 0.87 to 0.12)	0.77
PRS	0.3 (− 0.17 to 0.08)	0.2	0.052 (0.006 to 0.09)	0.028	0.0 (− 0.01 to 0.04)	0.91

*RR* recurrence rate, *PRS* prolapse-reduction suture

### Complications

One patient (1 %) had anal stenosis-requiring dilation. One patient (1 %) suffered from urinary retention. Although considerable time had elapsed between the procedure and the onset of urinary retention, no other cause could be found. One patient (1 %) reported mild faecal incontinence 7 months after the procedure.

## Discussion

While a variety of treatment options is available for grade I and II haemorrhoids, only SH and CH are considered to be a standard treatment for grade III disease, and just CH in the case of grade IV. In a meta-analysis [13], the RR for prolapse following CH is reported to be 4 %. However, both treatment options can be associated with extensive post-operative pain [5], faecal incontinence and anal stenosis [3]. Nonetheless, for most authors CH remains the standard treatment for advanced piles, because novel techniques including SH do not show the same effectiveness.

Although initial results from single centres have been promising [10, 12, 14–17], DG-HAL/RAR has not yet been adequately evaluated for grade III and IV haemorrhoids. The present study is the first international, multi-centre observational trial on the safety and effectiveness of DG-HAL/RAR. We observed no major intra- or post-operative complications. The overall complication rate was 8 %. In terms of prolapse recurrence, results achieved by CH at 12 months are slightly better than those recorded during our trial [2]. In terms of control of other symptom, results from our series of high-grade HAL/RAR patients were superior to those published for CH und SH [2].

Most studies for grade III and IV haemorrhoids focus on the recurrence of prolapse, although prolapse may not be the leading symptom in a significant number of patients. We believe that control of symptoms other than prolapse and patient satisfaction is more relevant in evaluating efficacy. In terms of pain, pruritus or bleeding, almost nine out of ten patients were treated successfully and satisfied after a follow-up of 12 months.

In most cases, the variation was due to the persistence or development (as a result of shrinkage of haemorrhoidal tissue) of skin tags, which subsequently required further treatment. This problem is also prevalent among patients having undergone CH and SH. In our series, 7 % of patients required rubber band ligation, sclerotherapy or haemorrhoidectomy within 12 months of undergoing DG-HAL/RAR and 8 % of patients received unspecified topical ointments, which we consider to be a low number. Taking all the above factors into account, we consider that our study demonstrates DG-HAL/RAR to be a safe and effective technique for grade III and IV haemorrhoids.

Furthermore, our study showed a low degree of post-operative pain. However, it should be noted that this study was not designed to assess post-operative pain for DG-HAL/RAR and, as such, pain assessment was only performed during the patient’s hospital stay. The greatest extent of pain was experienced immediately post-operatively, and the most frequent cause of pain was perianal thrombosis. As the median hospital stay was only 2 days and patients without post-operative pain would have been discharged early, the number of patients with higher pain levels may be over-represented. Despite this technicality, post-operative pain would appear to be quite low. It should be mentioned that some centres performed the DG-HAL/RAR as a day-case procedure, but others require an overnight stay due to reimbursement considerations. This may have more impact on the length of stay than pain alone.

For the first time in a study concerning DG-HAL/RAR, a multi-variate analysis was carried out to demonstrate the statistically significant influence of various factors on parameters of efficacy. The number of PRSs and in particular the number of ligations were shown to influence the recurrence of symptoms, whereas the only factor affecting the recurrence of prolapse was the grade of disease. It is interesting to note that the only factor significantly influencing patient satisfaction was the number of ligations. Our data show a trend towards five to seven ligations and three to four PRSs; however, as the number of PRSs and ligations depended on each patient’s vascularity as well as the surgeon’s opinion intra-operatively, and therefore varied considerably, it is difficult to establish the optimum number of ligations.

## Conclusion

HAL-RAR proved to be safe and effective in the present study, showing an acceptable prolapse RR of 11 % after 12 months for this series of high-grade haemorrhoid patients (59 % grade III, 41 % grade IV). Symptom control and patient satisfaction are high, resulting in approximately nine out of ten patients staying symptom-free and satisfied 1 year after surgery. The perioperative complications, which were low in number, were primarily cases of perianal thrombosis and post-operative bleeding. However, to answer the question of the optimum treatment modality (CH, SH and DG-HAL/RAR) for advanced haemorrhoidal disease, a controlled randomized trial would be needed. A multi-centre approach seems mandatory, because no single institution will be able to enrol enough patients or perform all three techniques with adequate experience. However, fully informed consent for such a trial may be problematic. In the absence of such a trial, the nine centres contributing to this study have chosen HAL-RAR as their first-line treatment for high-grade haemorrhoids owing to the perioperative benefits, very low number of complications, good symptom control and acceptably low RR.
